# Imaging mass spectrometry identifies prognostic ganglioside species in rodent intracranial transplants of glioma and medulloblastoma

**DOI:** 10.1371/journal.pone.0176254

**Published:** 2017-05-02

**Authors:** Leonardo Ermini, Elena Morganti, Alexander Post, Behzad Yeganeh, Isabella Caniggia, Michael Leadley, Claudia C. Faria, James T. Rutka, Martin Post

**Affiliations:** 1 Program in Physiology and Experimental Medicine, Peter Gilgan Centre for Research and Learning, Hospital for Sick Children, Toronto, Ontario, Canada; 2 The Lunenfeld-Tanenbaum Research Institute, Mount Sinai Hospital, Toronto, Ontario, Canada; 3 Institute of Medical Science, University of Toronto, Ontario, Canada; 4 Department of Physiology, University of Toronto, Ontario, Canada; 5 Division of Neurosurgery, Arthur and Sonia Labatt Brain Tumour Research Centre, Hospital for Sick Children, Toronto, Ontario, Canada; 6 Instituto de Medicina Molecular, Faculdade de Medicina, Universidade de Lisboa, Lisbon, Portugal; George Washington University, UNITED STATES

## Abstract

Matrix-assisted laser desorption ionization (MALDI) imaging mass spectrometry (MALDI-MSI) allows us to investigate the distribution of lipid molecules within tissues. We used MALDI-MSI to identify prognostic gangliosides in tissue sections of rat intracranial allografts of rat glioma and mouse intracranial xenografts of human medulloblastoma. In the healthy adult rodent brain, GM1 and GD1 were the main types of glycolipids. Both gangliosides were absent in both intracranial transplants. The ganglioside GM3 was not present in the healthy adult brain but was highly expressed in rat glioma allografts. In combination with tandem mass spectrometry GM3 (d18:1/C24:0) was identified as the most abundant ganglioside species in the glioma allotransplant. By contrast, mouse xenografts of human medulloblastoma were characterized by prominent expression of the ganglioside GM2 (d18:0/C18:0). Together, these data demonstrate that tissue-based MALDI-MSI of gangliosides is able to discriminate between different brain tumors and may be a useful clinical tool for their classification and grading.

## Introduction

Gangliosides are glycolipids containing ceramide and sialic oligosaccharides (named also glycosphingolipids). They are situated in the external leaflet of the cell membrane and are copiously expressed in the central nervous system. In tumor cells the rate of uptake and/or shedding of gangliosides in the microenvironment surrounding the cellular membrane is greatly increased [1]. GM3 and GD3 are the major gangliosides in embryonic brains. The expression of these simple gangliosides decreases with advancing brain development while that of complex gangliosides (GM1, GD1, and GT1) increases [2]. Similar to the embryonic brain, simple gangliosides (GM3 and GD3) are the most common gangliosides present on the cell surface in neoplastic tissues. In human gliomas, gangliosides have an altered conformation and concentration matched to normal grey and white matter of brain. The major gangliosides GM1, GD1a, and GT1b are markedly reduced in gliomas while gangliosides GM3 and GD3 prevail [3]. In human medulloblastoma, the most common malignant pediatric brain tumor, the main gangliosides, based on glycosphingolipid analysis of a medulloblastoma cell line, are GM2, GM3, and GD1a [4, 5]. In neoplastic tissues, the biological functions of gangliosides are influenced by changes of their structure [6, 7]. For example, in neuroblastoma, the short ganglioside GD2 containing the ceramide 16:0 has more immunosuppressive activity than GD2 gangliosides compromising longer ceramide chains (24:0 or 24:1), which are more abundant in non-pathological tissue [8]. These changes in ganglioside expression in tumours have led to their use as diagnostic and prognostic molecular markers for certain neural tumours [9–12]. However, classification, grading and prognosis of human brain tumours based on histological features of ganglioside expression have been limited by the lack of a method to visualize distinct ganglioside species. MALDI imaging mass spectrometry (MALDI-MSI) is a powerful technique to visualize the distribution of several type of molecules within tissues [13]. MALDI-MSI has extensively been used to visualize various lipids in tissues [14–21]. Cryosections are coated with a specific MALDI matrix and subsequently subjected to ionization thanks to a laser beam that moves across the tissue surface. Analyte ions are desorbed from the tissue sections and successive mass spectra are detected. The molecular distribution of the analytes as well as their intensity is then obtained as function of spatial coordinates. Several reports have shown the suitability of MALDI-MSI for analysis of ganglioside molecular species in brain tissues [20, 22–25], but this MSI technique has to our knowledge not been applied to brain tumors. Here we first investigated ganglioside metabolism and distribution in rat intracranial allografts of rat glioma using MALDI-MSI. The identity of discovered gangliosides was confirmed by LC-MS/MS analysis. To verify that MALDI-MSI of gangliosides could discriminate between different brain cancers we also analysed mouse intracranical xenografts of human medulloblastoma.

## Material and methods

### Cell lines and animals

Rat 9L gliosarcoma cell line [26] was generously donated by Dr. Roberto Diaz, Hospital for Sick Children, Toronto, Canada. The human medulloblastoma Daoy cell line [4] was kindly provided by Dr. Annie Huang, Hospital for Sick Children, Toronto, Canada. Athymic nude mice and Wistar rats were obtained from the Charles River Laboratories (Saint-Constant, QC).

### Tissue transplants

Animal studies were approved by the Institutional Animal Care Committee of the University of Toronto and the Hospital for Sick Children (Toronto, Canada) and performed according to their policies and regulations. The glioma and medulloblastoma cell lines were injected into the brain of Wistar rats or athymic nude mice using a stereotactic frame. Animals were operated under isoflurane anesthesia (3–4% in 1L O_2_ for induction and 1–2% in 1L O_2_ for maintenance) while meloxicam (5mg/Kg, subcutaneous) was given as analgesic. All animals received meloxican (5mg/Kg, subcutaneous) once daily for 48h after surgery to avoid any pain. Rodents were monitored daily for clinical signs of tumour growth, including hunched or abnormal posture, lack of grooming, weight loss exceeding 20% of body weight, anorexia, or abnormal ambulation. The presence of any of these findings was considered an endpoint and mice were euthanized using a CO_2_ chamber.

### MALDI-mass spectral imaging

#### Preparation of brain tissue sections

The quick frozen brain tissue block was mounted onto the specimen disc of a cryostat (Leica Microsystems, Richmond Hill, ON) using optimal cutting temperature (OCT) compound (Sakura Finetek, Torrance, CA). The brain sections were horizontally sliced at a thickness of 12 μm at -15°C and mounted onto indium tin oxide (ITO)-coated glass slides. A thin matrix layer was applied to the brain sections using an automated MALDI plate matrix deposition system (TM-Sprayer™, Leap Technologies, Carrboro, NC). A total of 5 mL of 9-aminoacridine (15 mg/mL in methanol; Sigma-Aldrich, St. Louis, MO) was sprayed per slide during 4 passes at 80°C with a velocity of 400 mm/min and a line spacing of 3 mm.

#### MALDI imaging mass spectrometry

The acquisition of the images was performed as previously described [17, 27, 28]. A time-of-flight tandem mass spectrometer (SCIEX TOF/TOFTM 5800 System; SCIEX, Vaughan, ON) was used to acquire the images. MALDI mass spectra were obtained using a Nd:YAG laser (349 nm) at 3 ns pulse width and 400 Hz firing rate. To install the ITO-coated glass slides in the ionization chamber, we used a special holder (SCIEX) having concavities. The data were acquired in the negative-ion reflector mode using an external calibration method. The external calibration gangliosides (Avanti Polar Lipids, Alabaster, AL; Sigma-Aldrich, St Louis, MO) were deposited on the ITO-coated slides to minimize mass shift (see S1 Fig). A total of 200 laser shots per point were irradiated (1 s/point) and the interval between data points are 75 μm. The mass spectrometric data were processed using a specialized script of Analyst software (SCIEX) at a mass resolution of 0.1 amu and images were visualized using TissueView software (SCIEX). The imaging experiments were repeated with 3 different intracranial transplanted mice and rat brains. The gangliosides identified by MALDI-MSI are listed in S1 Table.

### Immunohistochemistry

Cryostat sections of 10-μm thickness were cut from mouse and rat brains and stained for Cholera Toxin B (CTB) and Neuropilin-1 (NRP-1) as described previously [29]. Briefly, tissue sections were rehydrated and blocked for one hour at room temperature followed by incubation with either the biotin-conjugated CTB subunit (1:500; Sigma, St Louis, MO) or rabbit anti-NRP1 (1:1000; gift of Dr. D.D. Ginty, Harvard University) antibody overnight at 4°C in a humidified chamber. The primary antibody was detected by using a biotinylated secondary anti-rabbit IgG (Jackson ImmunoResearch Laboratories). The biotin complexes were visualized using an ABC kit (Vector laboratories, Burlington, ON). As a negative control, sections were processed as above but CTB subunit or primary antibody was omitted.

### Rat brain glycosphingolipid extraction

Tissue sections were sliced at a thickness of 80 μm at -15°C in cryostat and mounted onto superfrost microscope slides (Fisher Scientific, Ottawa, ON). Squares of 2 mm of the pathological and physiological brain area were cut out, weighed, transferred to siliconized screw capped glass extraction tubes and homogenized using a Polytron tissue grinder (three 10-s bursts with 20-s intervals) in 4 volumes (4 ml/g wet weight) water. To the homogenate were then added 2.67 volumes (based on the total aqueous volume) of ambient temperature (RT) methanol and 1.33 volumes of chloroform, tubes capped and mixed vigorously. The mixture was centrifuged at 450 x *g* at RT for 15 min and the supernatant was transferred to a fresh screw-capped tube. A small (0.173) volume of water was added and the mix was centrifuged again at 450 x *g* at RT for 15 min. The supernatant was then loaded on C18 Sep-Pak. The column was washed with 3 ml each of the following: (i) chloroform-methanol-water (2:43:55) and (ii) methanol-water (1:1). The gangliosides were eluted with 3 ml of methanol, collected into a fresh screw capped tube and evaporated to dryness under a stream of dry nitrogen at 45 °C. The gangliosides were then re-dissolved in 2.5 volumes of methanol, based on the original tissue weight adjusting for recovery of “recovered extract volume” and subjected to LC-MS/MS analysis.

### LC-MS/MS analysis

LC-MS/MS was performed on an Agilent 1200 HPLC (Agilent Technologies: Santa Clara, California, USA) and a Sciex API4000 Mass Spectrometer (Sciex, Vaughan, ON). Chromatography ran at a flow rate of 500 μL/min on an Agilent SB-Phenyl 3.5μ, 3.0x50 mm column. The mobile phase consisted of A = Water and B = 10/45/45 Water/Methanol/Acetonitrile, both A and B containing 10 mm Ammonium Acetate. The mass spectrometer was operated in negative ESI mode with a source temperature of 600°C, an ESI voltage of -4,500, DP of -40, and EP of -10 volts. Data was acquired in by single MS with Q1 scanning from 1,100 m/z to 1,600 m/z. LC/MS/MS precursor ion scanning of 290 m/z Q3 and 1,200 m/z to 1,400 m/z Q1 was performed with the same MS parameters as above and additional settings of -50 CE (N_2_ gas) and -15 CXP volts. Data handling was performed using Sciex Analyst 1.6 software.

## Results

**Fig 1:**
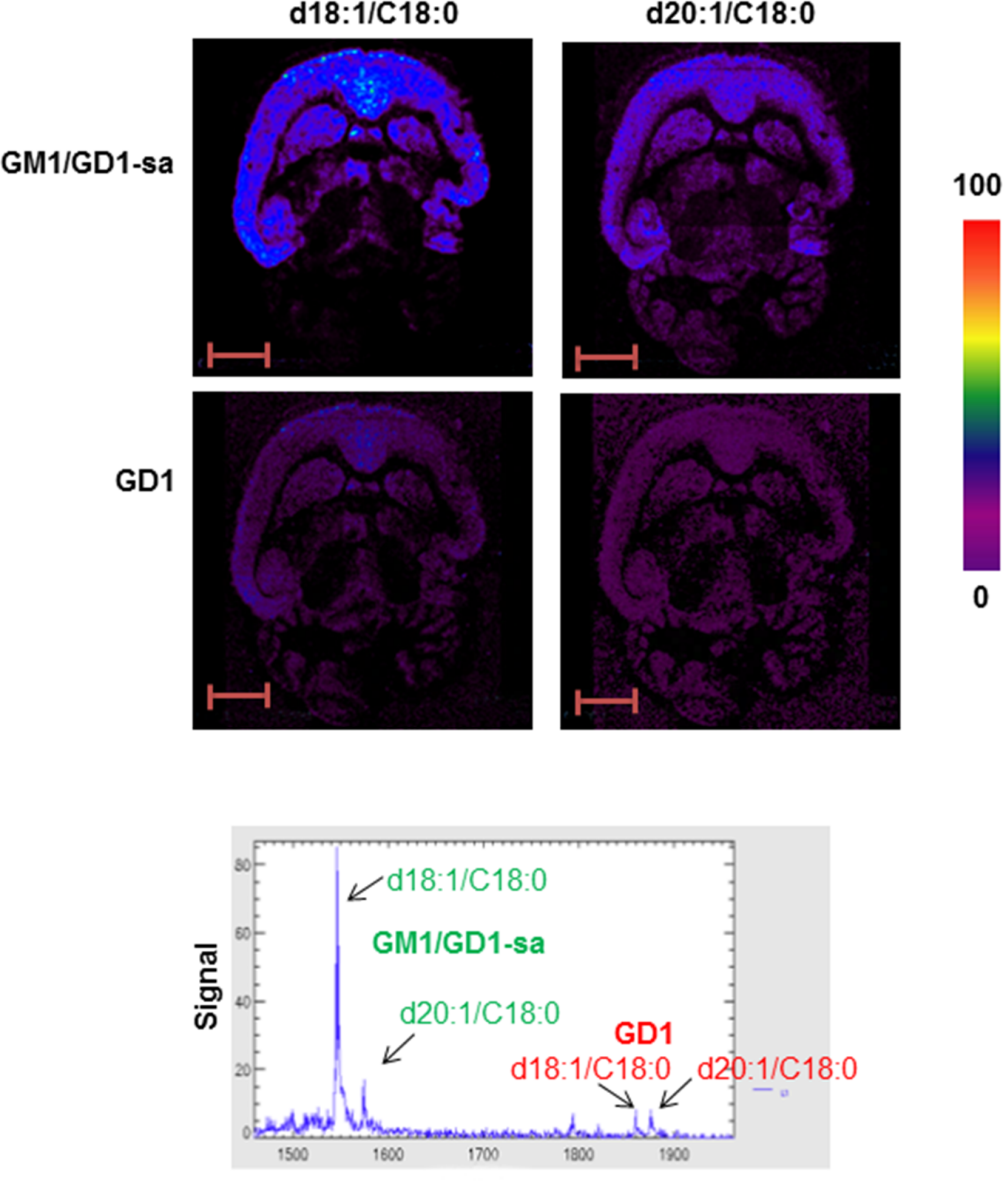
MALD-MSI of GM1 and GD1 in adult rat brain. MSI and mass spectra of GM1/GD1-sialic acid (H-) d18:1 (m/z 1545) and 20:1 (m/z 1573), GD1 ([M-H]2-) d18:1 (m/z 1874) and 20:1 (m/z 1886) in healthy adult Wistar rat brain. Intensities of the ions are represented in color based on the intensity scale provided. Arrows indicate the peaks visualized in MSI. GD1-sa = GD1-sialic acid.

**Fig 2:**
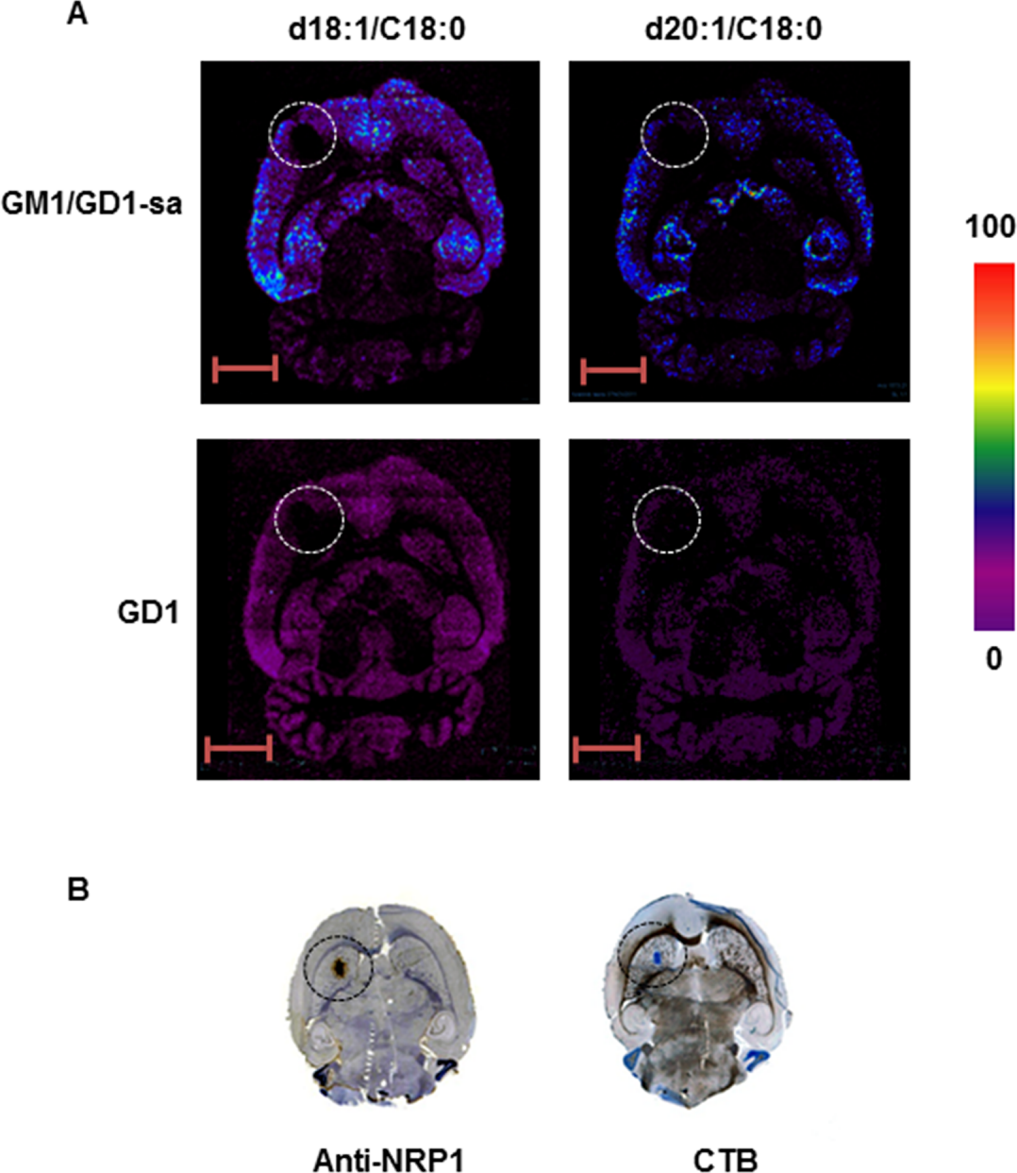
MALD-MSI of GM1 and GD1 in intracranial allografts of rat glioma. (A) MSI of GM1/GD1-sialic acid (H-) d18:1 (m/z 1545) and 20:1 (m/z 1573), GD1 [M+Na-2H] d18:1 (m/z 1874) and 20:1 (m/z 1886). Intensities of the ions are represented in color based on the intensity scale provided. (B) Distribution of GM1 and NRP-1 by immunostaining with CTB and anti-NRP1 antibodies, respectively, in intracranial allografts of rat glioma. Circles indicate the position of the xenograft. Scale bar: 5 mm. GD1-sa = GD1-sialic acid.

We first investigated the expression and distribution of GM1 and GD1 in brains of naïve adult Wistar rat using MALDI-MSI. The GM1 (d18:1/C18:0; m/z 1545; M-H) and (d20:1/C18:0; m/z 1573; M-H) gangliosides were present throughout the cerebral cortex (Fig 1). Expression of GD1 (d18:1/C18:0; m/z 1874; [M+Na-2H] and d20:1/c18:0; m/z 1886; [M+Na-2H]) gangliosides in the cortex overlapped with that of the GM1 (d18:1/C18:0) ganglioside. Analysis of our GD1 standards confirmed that GD1 gangliosides can lose their sialic acid moiety in the negative reflectron ion mode during MALDI-MSI [22] and that the resulting GD1 derivatives ionize at the same mass as GM1 (see S2 Fig). The distribution of GM1 in the brain was also identified by staining with the B subunit of cholera toxin (Fig 2B), which binds to GM1 [30]. The CTB staining revealed that GM1 was present in the subcortical white matter and less pronounced in the cortex. Thus, it is plausible that the MALDI-MSI detection of GM1 in the cortex is partially due to GD1 fragmentation. Also, the GD1 gangliosides are particularly abundant in the cortex. Therefore, we have labelled the peaks at 1545 and 1573 m/z as GM1/GD1-sialic acid (GM1/GD1-sa). Moreover, changing the scale and intensity of the ions revealed that GM1 also localized to the white matter (see S3 Fig).

The localization of the GM1 and GD1 gangliosides was not altered in the areas of rat brains containing an intracranial allograft of glioma (Fig 2A). However, both types of ganglioside were absent in the tumor (Fig 2A, circle). Negative CTB staining of the rat glioma (Fig 2B) corroborated our GM1 findings using MALDI-MSI (Fig 2A). The glioma cells were positively identified by neuropilin-1 (NRP-1) immunohistochemistry (Fig 2B). NRP-1 is a cell surface glycoprotein that is highly expressed in many cancers, including glioma [31] and medulloblastoma [32].

**Fig 3:**
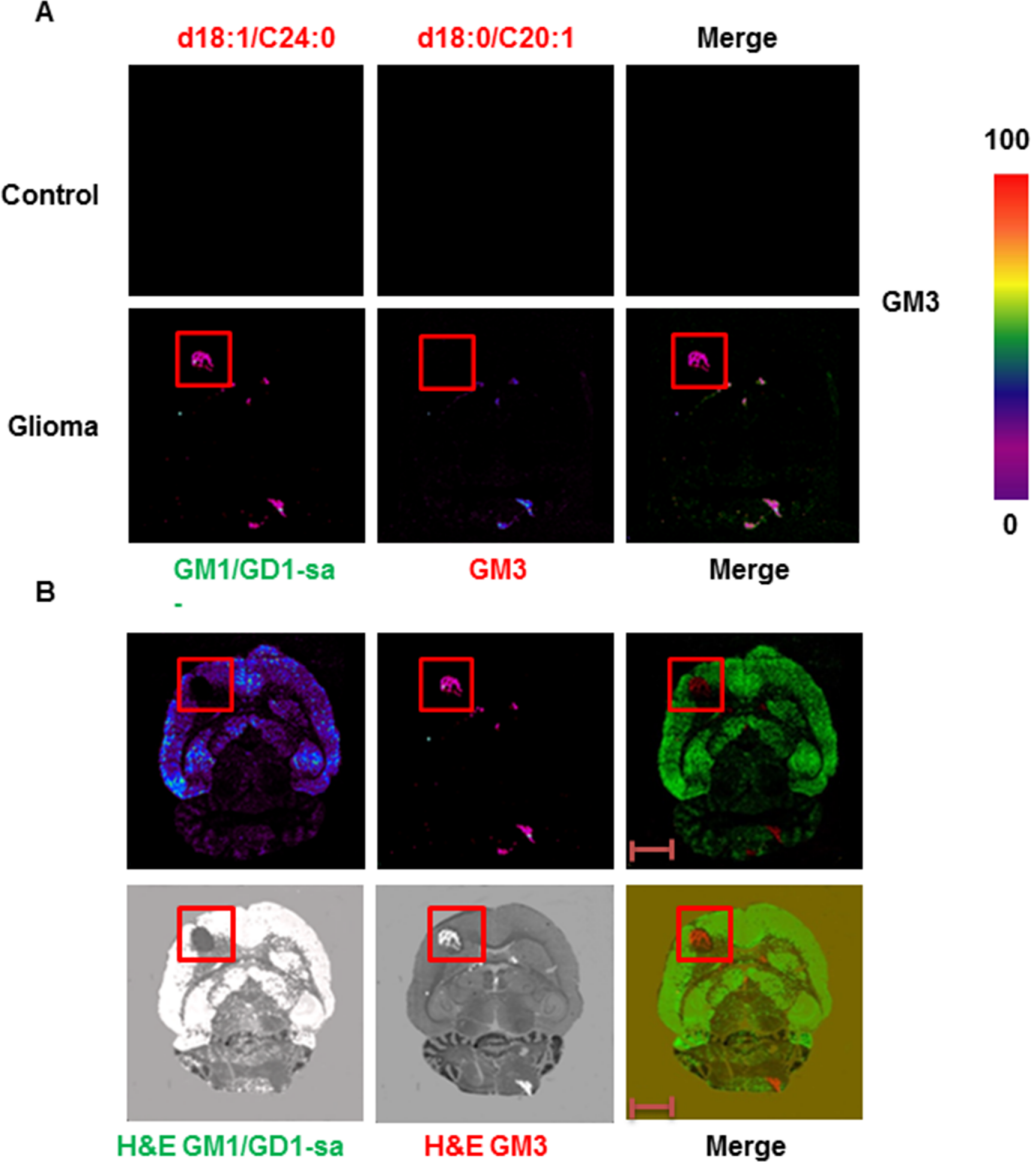
MALD-MSI of GM3 in intracranial allografts of rat glioma. (A) MSI of GM3 (H-) d18:1 24:0 (m/z 1264) and d18:0 20:1 (m/z 1208) in intracranial allografts of rat glioma. Intensities of the ions are represented in color based on the intensity scale provided. (B) Molecular histology distribution of GM1/GD1-sialic acid (d18:1/C18:0) and GM3 (d18:1/C24:0) in intracranial allografts of rat glioma. Red squares indicate the position of the glioma xenograft. Scale bar: 5 mm. GD1-sa = GD1-sialic acid.

The control rat brains and intracranial allografts of rat glioma were also analysed for the presence of GM2 and GM3. While the GM2 (d18:0/c18:0; m/z 1385; [M-H]) ganglioside was not detected in the control and allograft brain sections (S4 Fig), GM3 with a d18:1/C24:0 ceramide (m/z 1264; M-H) exclusively localized to the glioma transplant area (Fig 3A). Specifically, merging MALDI-MSI and H&E strongly demonstrated the unique expression of GM3 (d18:1/C24:0) in the glioma allotransplant (Fig 3B). In addition, merging MALDI-MSI with H&E highlighted the absence of GM1 gangliosides from the glioma allograft (Fig 3B).

We confirmed the MALDI-MSI data by LC-MS/MS analysis. The glioma allograft and a comparable non-pathological area were excised from 80-μm thick brain cryosections (S5A Fig) and subjected to glycosphingolipid extraction and detection by tandem mass spectrometry. The LC-MS/MS analysis verified the change in distribution from GM1 towards GM3 gangliosides in the glioma allograft compared to non-pathological brain tissue (S5B and S5C Fig). The most representative GM3 species in the glioma was GM3 (d18:1/C24:0; m/z 1264). Precursor ion scanning for m/z 290 fragment in negative mode confirmed the ganglioside identity of this mass peak. Moreover, mass spectral comparison between GM1/GM3 standards and glioma glycosphingolipid extract validated that the GM3 species in the cancer contained the ceramide d18:1/C24:0 (S5D Fig).

**Fig 4:**
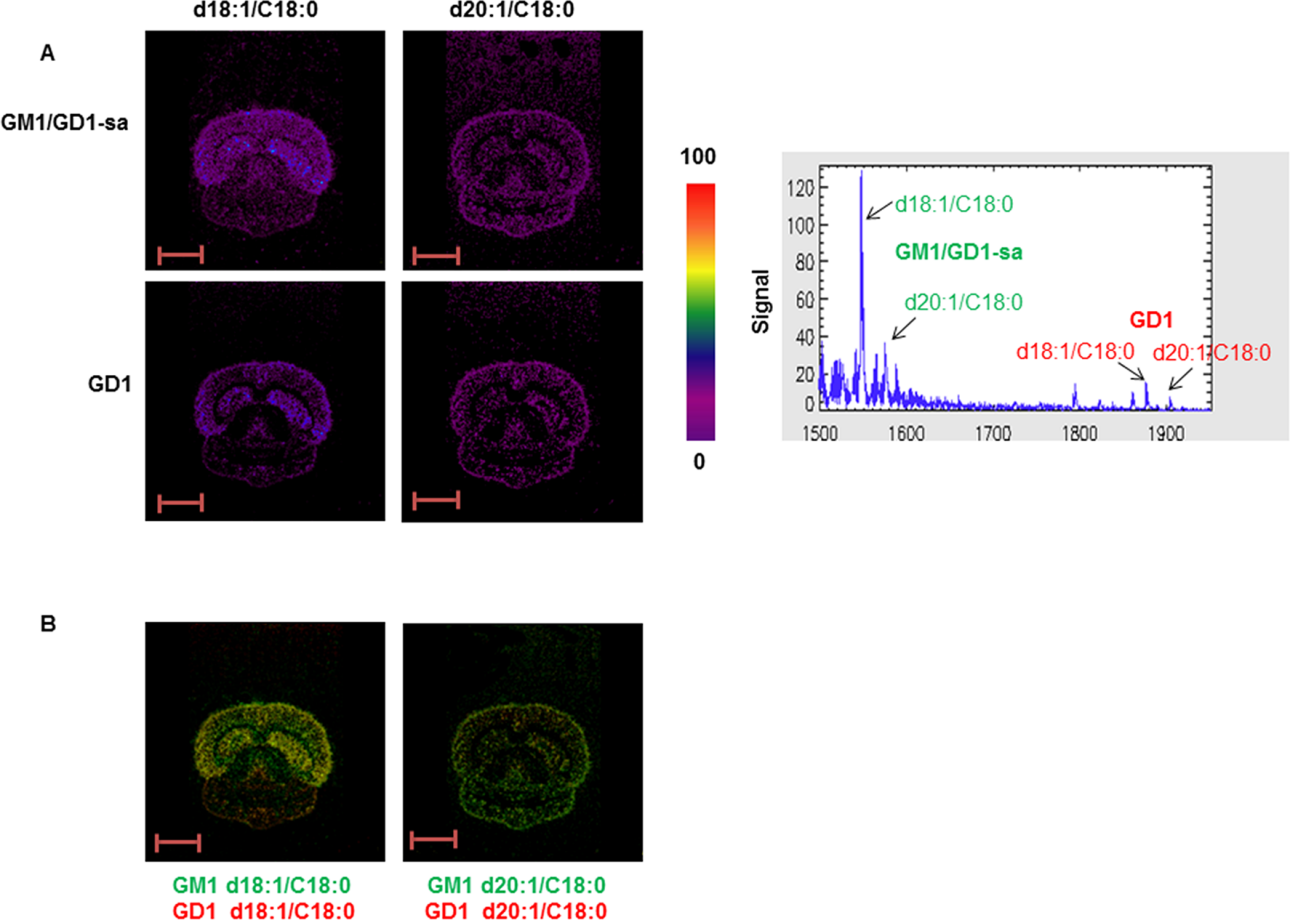
MALD-MSI of GM1 and GD1 in adult mouse brain. (A) MSI and mass spectra of GM1/GD1-sialic acid (H-) d18:1 (m/z 1545) and 20:1 (m/z 1573), GD1 [M+Na-2H] d18:1 (m/z 1874) and 20:1 [M+K-2H] (m/z 1902). Intensities of the ions are represented in color based on the intensity scale provided. Arrows indicate the peaks visualized in MSI. (B) Overlap of the distribution of GM1/GD1-sialic acid and GD1 species in healthy adult mouse brain. Scale bar: 4 mm. GD1-sa = GD1-sialic acid.

**Fig 5:**
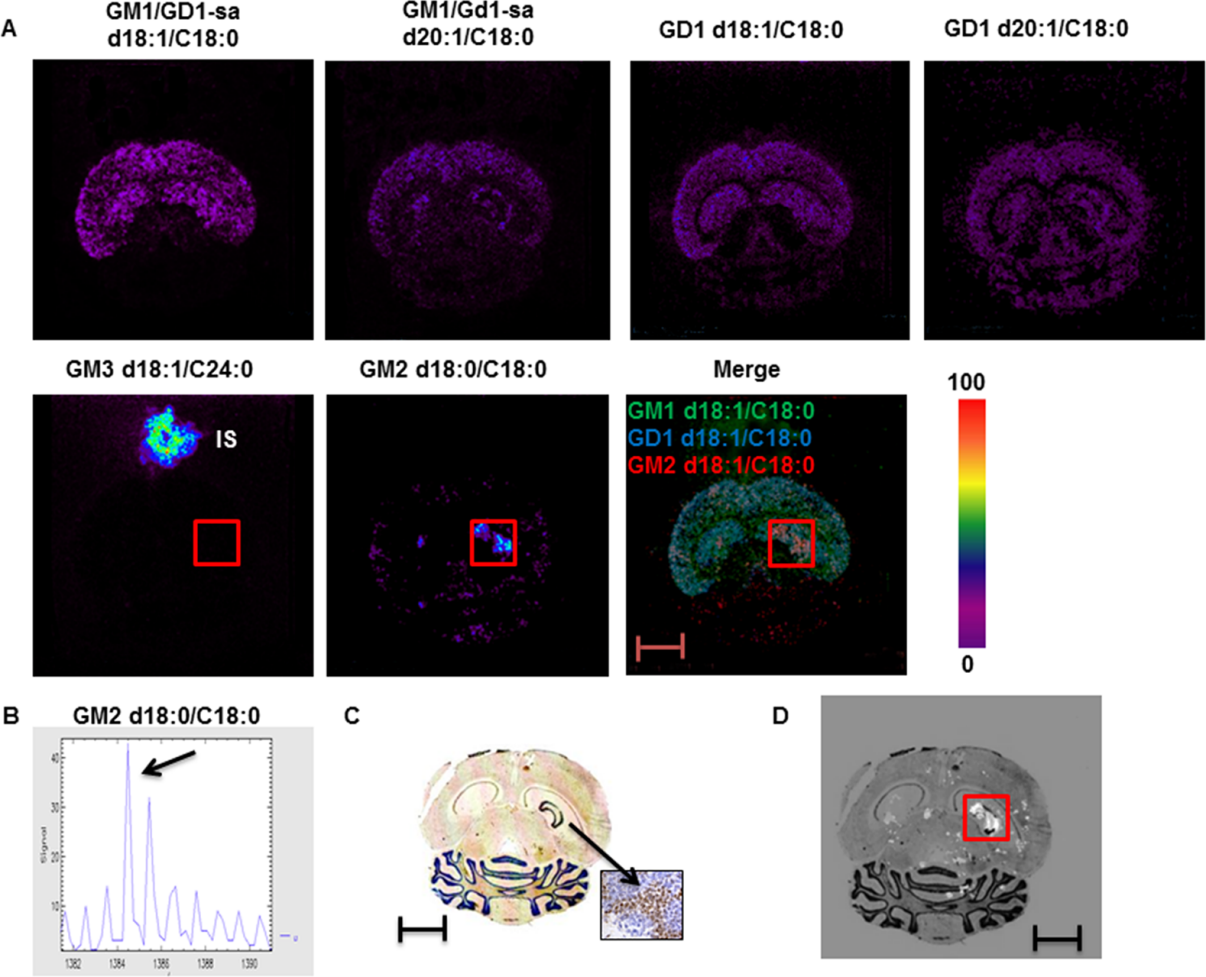
MALD-MSI of gangliosides in intracranial xenografts of human medulloblastoma. (A) MSI of GM1/GD1-sialic acid (H-) d18:1 (m/z 1545) and 20:1 (m/z 1573), GD1 ([M-H]2-) d18:1 (m/z 1874) and 20:1 (m/z 1902), GM3 (H-) d18:1 c24:0 (m/z 1264) and GM2 (H-) d18:0 c18:0 (m/z 1385) in intracranial xenografts of human medulloblastoma. Intensities of the ions are represented in color based on the intensity scale provided. (B) Mass spectra of GM2 (d18:0/C18:0) in intracranial xenografts of human medulloblastoma. (C) Distribution of Neuropilin-1 by immunostaining with anti-NRP1 antibodies in intracranial xenografts of human medulloblastoma. The insert is a magnification 40 x of the glioma area. (D) Molecular histology distribution of GM2 (d18:0/C18:0) in intracranial xenografts of human medulloblastoma. Squares indicate the position of the medulloblastoma graft. Scale bar: 4 mm. GD1-sa = GD1-sialic acid.

We then analyzed intracranical mouse xenografts of human medulloblastoma using the same approach. We initially examined the expression and distribution of GM1 and GD1 in the naïve adult mouse brain and found that the distribution of these two gangliosides (Fig 4) matched that of the rat brain (Fig 1). The distribution of these two ganglioside species was maintained in the mouse brains with the intracranial xenografts of human medulloblastoma (Fig 5A). The intracranial xenografts of human medulloblastoma were then examined by MALDI-MSI for the presence of GM2 and GM3. By contrast to glioma allografts, ganglioside GM3 (d18:1/C24:0) was not detected in the medullablastoma xenograft (Fig 5A). Only the internal standard (IS) was visible. However, MALDI-MSI revealed a GM2 species with a d18:0/C18:0 ceramide (m/z 1385; M-H) that exclusively localized to the medulloblastoma xenograft area (Fig 5A and 5B). Merging of MALDI-MSI and H&E confirmed the expression of GM2 (d18:0/C18:0) in the medulloblastoma xenograft (Fig 5D). The medulloblastoma cells were positively identified by NRP-1 immunohistochemistry (Fig 5C).

## Discussion

Here we show the expression and distribution of various types of ganglioside in normal adult rodent brains and in intracranial allo- and xenografts of rat glioma and human medulloblastoma, respectively, using MALDI-MSI. In line with previous reports, the major gangliosides within the normal rodent brain are GM1 and GD1 [2]. GD1 can lose its sialic acid moiety in the negative reflectron ion mode during MALDI-MSI and, in that instance, it will be detected as a GM1 species [22]. The observed similar distribution of GM1 and GD1 gangliosides in the rat and mouse brain and the white matter staining of GM1 with CTB supports the idea that the GM1 signals in the cortex is partially derived from GD1 gangliosides.

Abnormal ganglioside expression in brain cancer and the roles of these glycosphingolipid in brain tumor development, progression, and treatment have been reported in several studies [33, 34]. By MALDI-MSI we identified GM3 as the predominant ganglioside species in the rat glioma allograft. GM3 is the simplest ganglioside oligosaccharide and serves as a precursor for most of the more complex ganglioside species including GD3, GM2 and GD2 [35]. GM3 is strongly expressed in the human cerebellum between 21 and 25 weeks of gestation, a period of intense cell proliferation. At 27 weeks, there is a significant reduction in its expression and GM3 is virtually absent in the brain by the second postnatal month [36]. In contrast, GM3 is overexpressed in neoplastic tissues and is the most common type of ganglioside present in tumor cells [37]. The role of GM3 has mostly been studied in the apoptosis and drug resistance of neoplastic cells [38–41]. Whether the GM3 (d18:1/C24:0) species plays a role in these processes is unknown as in previous studies the chemical structure of GM3 was not determined [42].

The ganglioside profile in brain tumors has been shown to correlate with tumor histopathological origin, malignancy grade, invasiveness, and progression [43]. In the present study, we found that MALDI-MSI screening for gangliosides was able to distinguish between two types of brain cancer. MALDI-MSI identified a medulloblastoma xenograft by expression of the ganglioside GM2 while glioma allografts expressed GM3. MALDI-MSI did not identify any GM3 species in the medulloblastoma xenograft, while GM3 has been reported to make up approximately 13% of total gangliosides of the human Daoy medulloblastoma cell line [4]. It is well known that cells *in situ* have different expression patterns compared to cultured cells and thus it is very likely that GM3 is not expressed in the Daoy-derived medulloblastoma *in situ*. Alternatively, the GM3 concentration was under the limit of detection of MALDI-MSI or the irradiated area was too small. It has been shown that the amount of analyte detected by MALDI-MSI decreases as the square of the spatial resolution [44]. Normally, GM2 is a minor ganglioside in most tissues but gets highly expressed in certain cancer cells [45], including the Daoy medullablastome cell line [4]. We were not able to confirm the identity of the GM2 species by LC-MS/MS in the intracranical medulloblastoma xenograft because of the small amount of tissue available. However, our MALDI-MSI observation of GM2 being one of the prominent gangliosides in the intracranial medulloblastoma xenograft agrees with previous findings [4].

In conclusion, molecular imaging is a technique that permits histological investigation of molecules such as gangliosides that are involved in the structural and functional aberrations of cancer cells. Here we provide evidence that MALDI-MSI is able to discriminate between various molecular species of gangliosides expressed by different brain tumors. In the future, it might be possible to apply mass spectrometry imaging for routine clinical differentiation of brain tumors.

## Supporting information

S1 Table Principal ganglioside species identified by MALDI-MSI. (DOCX)

S1 Fig MALD-MSI of GM1/GD1-sialic acid and GM3 in intracranial allografts of rat glioma. MSI (A) and mass spectra (B) of GM1/GD1-sialic acid (d18:1/C18:0) and GM3 (d18:1/C24:0) in intracranial allografts of rat glioma. BF = bright field. Arrows indicate the peaks visualized in IMS. IS: Internal Standard.
(TIF)

S2 Fig MALDI-MSI of GM1/GD1-sialic acid and GD1 in normal mouse brain. (A) MSI and mass spectra of GM1/GD1-sialic acid (d18:1/C18:0) and (B) GD1 (d18:1/C18:0) in normal mouse brain. IS: Internal Standard.
(TIF)

S3 Fig MALDI-MSI of GM1/GD1-sialic acid in normal rat and mouse brain. The scale and intensity of ions has been changed to visualize the GM1 distribution in the white matter.
(TIF)

S4 Fig MALD-MSI of GM3 and GM2 in intracranial allografts of rat glioma. (A) MSI of GM3 [d18:1/c24:0 - (m/z 1264)] and GM2 [d18:0/c18:0 - (m/z 1385)]. (B) Mass spectra of GM2 (d18:0/c18:0) was negative. Arrow indicate the peak of GM2 (d18:0/c18:0) visualized in IMS.
(TIF)

S5 Fig Mass spectrometry analysis of nonpathological rat brain tissue (blue) and rat glioma allograft (red). (A) Areas cut from thick brain cryosections are marked by squares. (B) Overlay of GM1 1545 m/z from nonpathological brain tissue vs glioma allograft. (C) Overlay of GM3 1264 m/z from nonpathological brain tissue vs glioma allograft. (D) Mass spectrometric profile of GM1 and GM3 standards.
(TIF)
