# Supplementary material for: Imaging mass spectrometry identifies prognostic ganglioside species in rodent intracranial transplants of glioma and medulloblastoma
Source: PLoS One. 2017 May 2;12(5):e0176254. doi: 10.1371/journal.pone.0176254 (PMC5413052; doi:10.1371/journal.pone.0176254)
Supplement: S1 Table — (DOCX) [file pone.0176254.s001.docx]

| Gangloside | Negative Ions | Experimental Mass  (m/z) | Theoretical Mass  (m/z) | Error (m/z) |
| --- | --- | --- | --- | --- |
|  |  |  |  |  |
| GM1/GD1-sialic acid (d18:1/18:0) | M-H | 1545.4 | 1544.9 | 0.5 |
| GM1/GD1-sialic acid (20:1/18:0) | M-H | 1573.5 | 1572.9 | 0.6 |
| GD1 (18:1/18:0) | M+K-2H | 1874.5 | 1874.1 | 0.4 |
| GD1 (20:1/18:0) | M+Na-2H | 1886.5 | 1886 | 0.5 |
| GD1 (20:1/18:0) | M+K-2H | 1902.3 | 1902.1 | 0.2 |
| GM2 (18:0/18:0) | M-H | 1385.4 | 1384.7 | 0.7 |
| GM3 (18:0/20:1) | M-H | 1208.3 | 1207.8 | 0.5 |
| GM3 (18:1/24:0) | M-H | 1264.1 | 1263.8 | 0.3 |

**S1 Table. Principal ganglioside species identified by MALDI-MSI**
