# Supplementary figures and images for: Imaging mass spectrometry identifies prognostic ganglioside species in rodent intracranial transplants of glioma and medulloblastoma
Source: PLoS One. 2017 May 2;12(5):e0176254. doi: 10.1371/journal.pone.0176254 (PMC5413052; doi:10.1371/journal.pone.0176254)

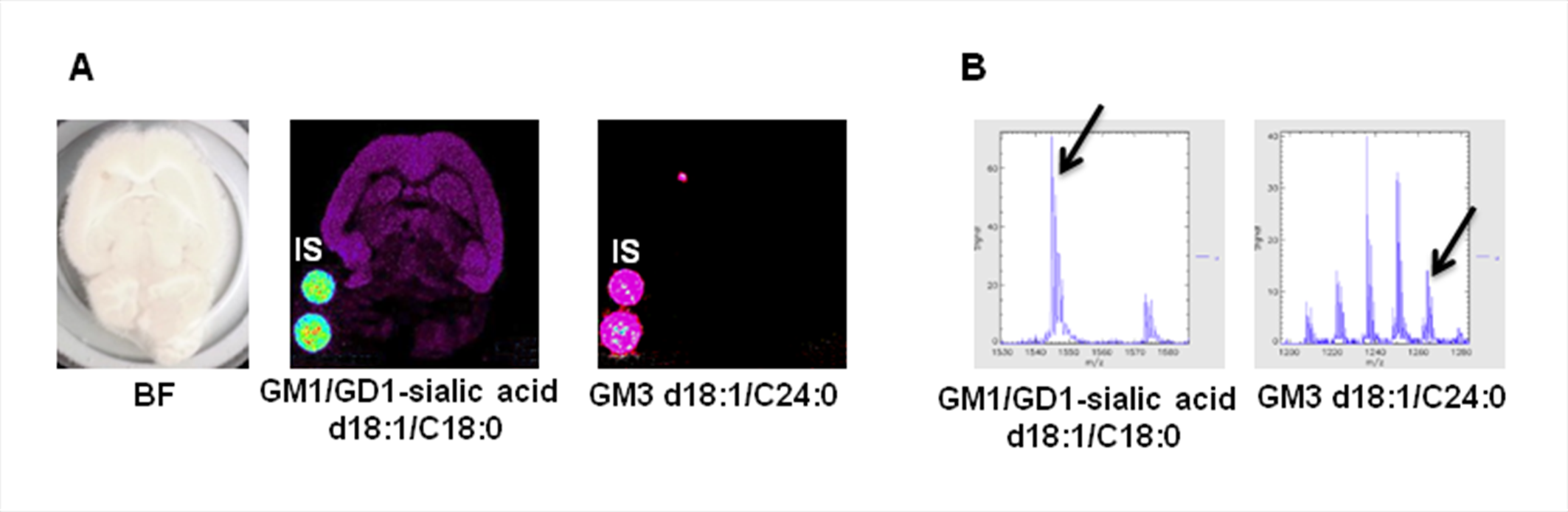

Supplement: S1 Fig — MSI (A) and mass spectra (B) of GM1/GD1-sialic acid (d18:1/C18:0) and GM3 (d18:1/C24:0) in intracranial allografts of rat glioma. BF = bright field. Arrows indicate the peaks visualized in IMS. IS: Internal Standard. (TIF) [file pone.0176254.s002.tif]

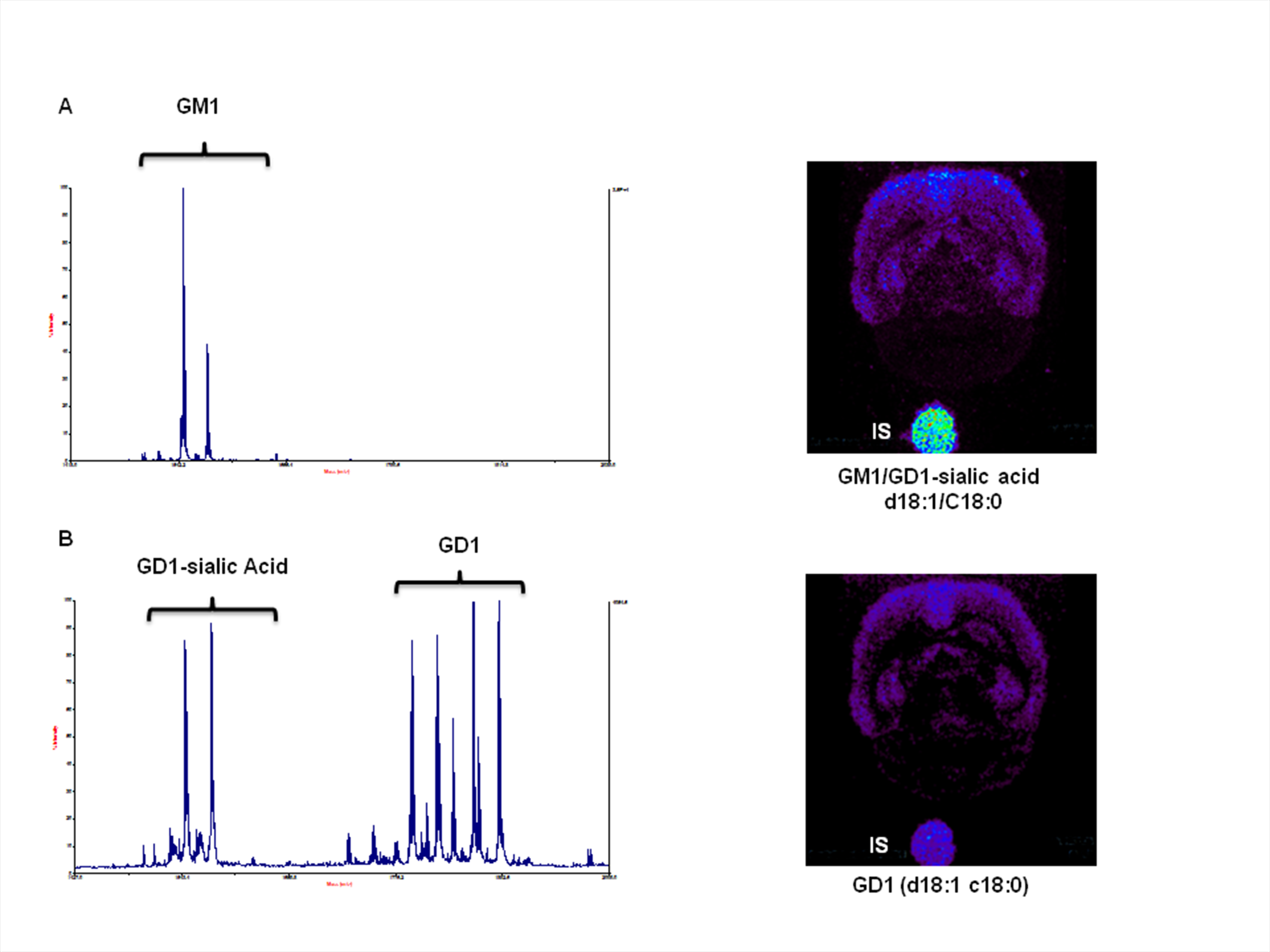

Supplement: S2 Fig — (A) MSI and mass spectra of GM1/GD1-sialic acid (d18:1/C18:0) and (B) GD1 (d18:1/C18:0) in normal mouse brain. IS: Internal Standard. (TIF) [file pone.0176254.s003.tif]

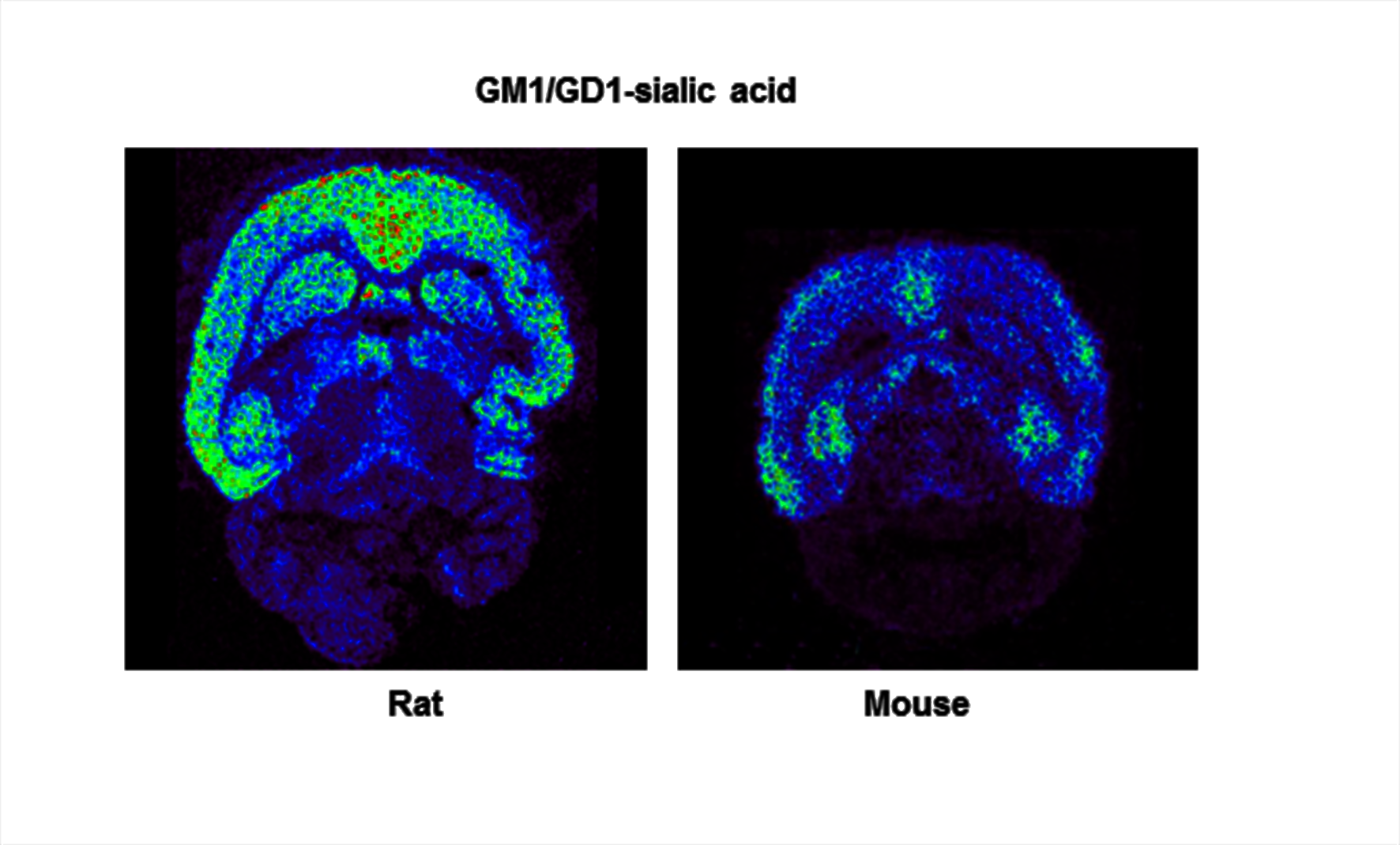

Supplement: S3 Fig — The scale and intensity of ions has been changed to visualize the GM1 distribution in the white matter. (TIF) [file pone.0176254.s004.tif]

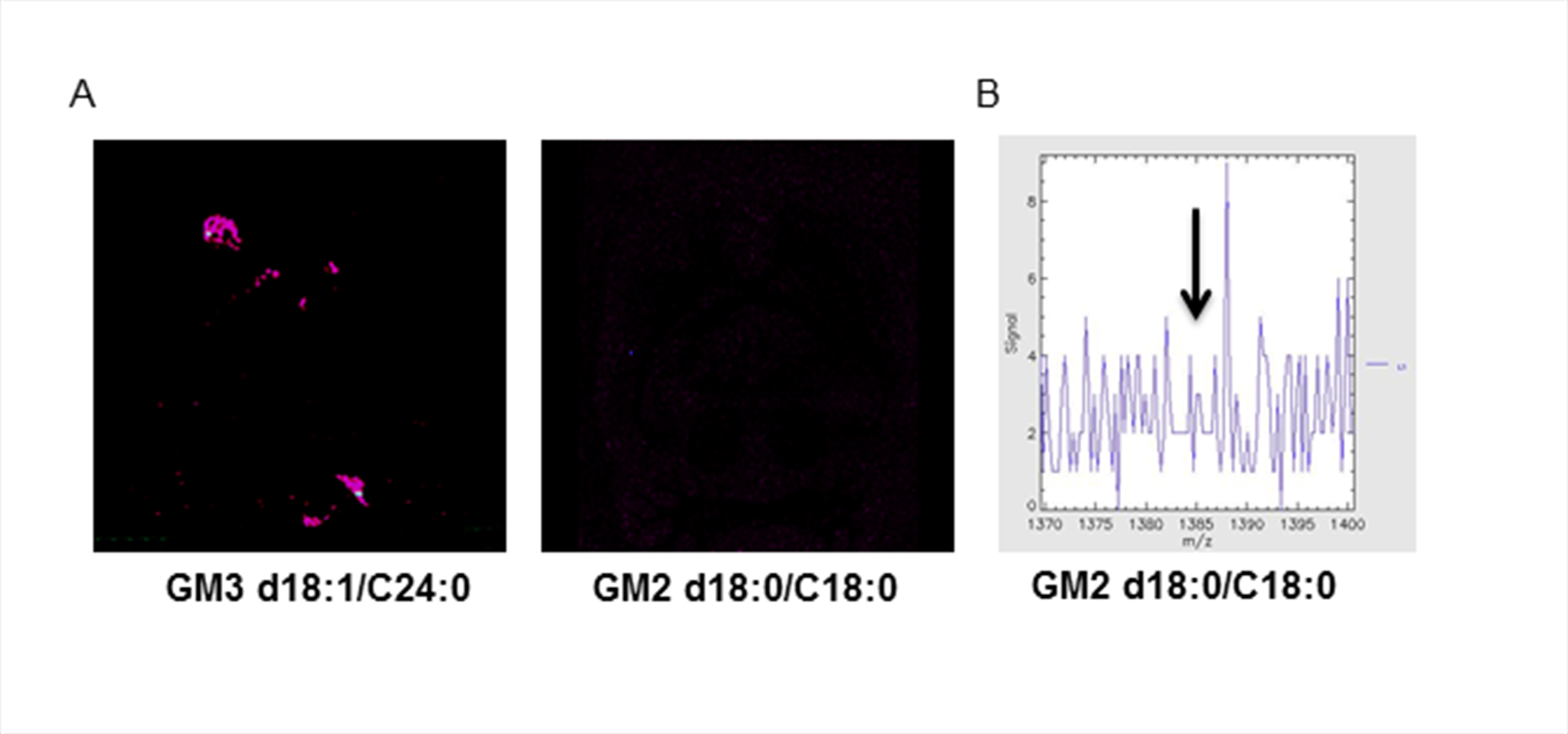

Supplement: S4 Fig — (A) MSI of GM3 [d18:1/c24:0 - (m/z 1264)] and GM2 [d18:0/c18:0 - (m/z 1385)]. (B) Mass spectra of GM2 (d18:0/c18:0) was negative. Arrow indicate the peak of GM2 (d18:0/c18:0) visualized in IMS. (TIF) [file pone.0176254.s005.tif]

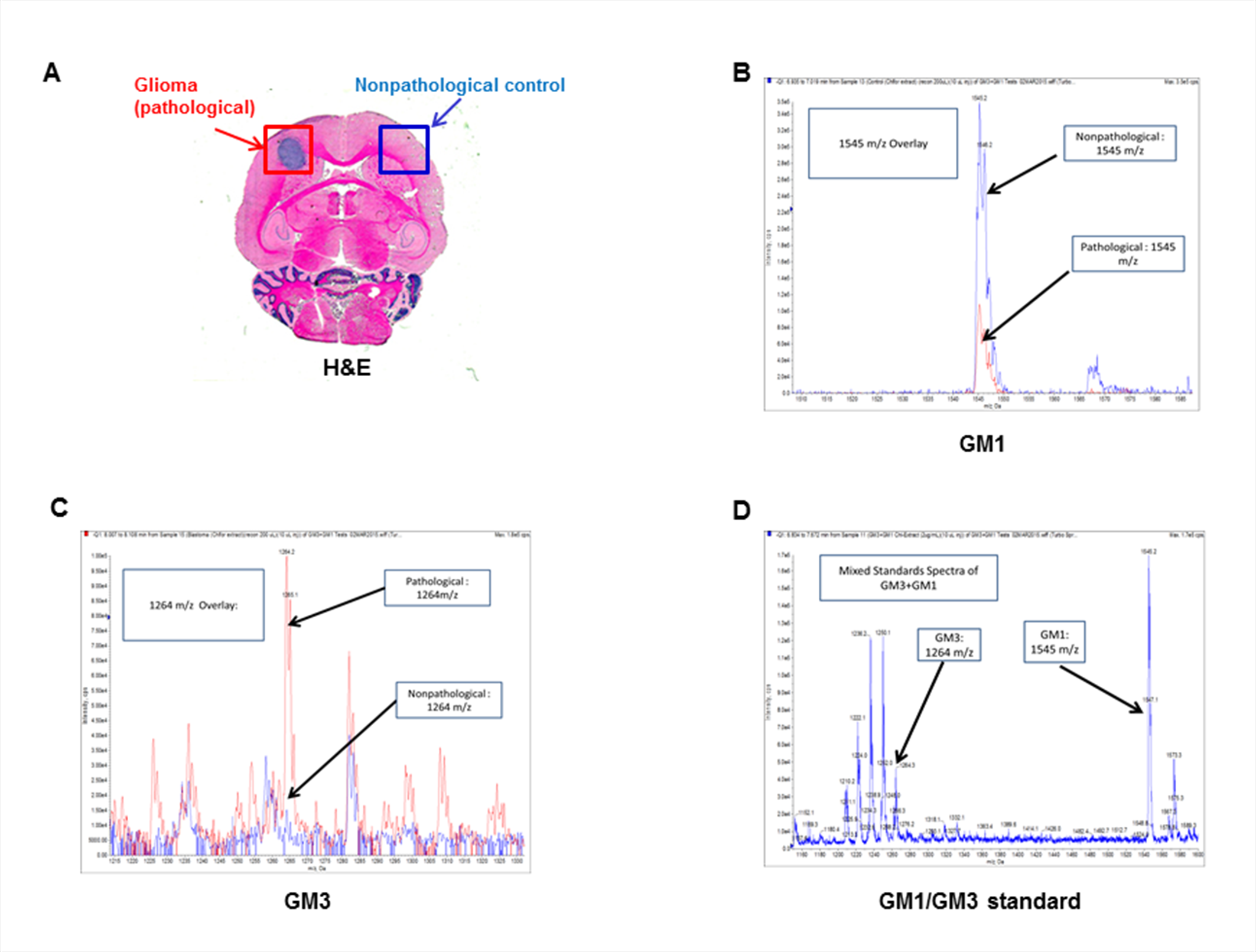

Supplement: S5 Fig — (A) Areas cut from thick brain cryosections are marked by squares. (B) Overlay of GM1 1545 m/z from nonpathological brain tissue vs glioma allograft. (C) Overlay of GM3 1264 m/z from nonpathological brain tissue vs glioma allograft. (D) Mass spectrometric profile of GM1 and GM3 standards. (TIF) [file pone.0176254.s006.tif]
